# Quality of life among non-metastatic breast cancer survivors in Botswana

**DOI:** 10.1007/s11764-025-01895-1

**Published:** 2025-09-20

**Authors:** Rachel B. Wagner, James R. Wester, Bosa Motladiile, Isaac Nkele, Nkhabe Chinyepi, Gontse Tshisimogo, Peter Vuylsteke, Shahin Lockman, Scott Dryden-Peterson, Racquel E. Kohler

**Affiliations:** 1 Center for Cancer Health Equity, Rutgers Cancer Institute, 120 Albany St, New Brunswick, NJ 08901, USA; 2 Department of Surgery, University of Pennsylvania, 3400 Spruce St, Philadelphia, PA 19104, USA; 3 Botswana Harvard Health Partnership, Plot No. 1836 North Ring Rd, Gaborone, Botswana; 4 Department of Surgery, University of Botswana, Plot 4775 Notwane Rd, Gaborone, Botswana; 5 Botswana Ministry of Health and Wellness, Plot 8WV5+J79, Nelson Mandela Dr, Gaborone, Botswana; 6 Department of Internal Medicine, University of Botswana, Gaborone, Botswana; 7 Division of Infectious Diseases, Brigham and Women’s Hospital, 75 Francis St, Boston, MA 02115, USA; 8 Department of Medicine, Harvard Medical School, 25 Shattuck Street, Boston, MA 02115, USA

**Keywords:** Breast cancer, Mastectomy, Quality of life, Sub-Saharan Africa

## Abstract

**Purpose:**

This study explored quality of life (QOL) experiences after non-metastatic breast cancer treatment, especially related to mastectomy, among survivors in Botswana.

**Methods:**

We conducted 23 qualitative interviews in Setswana with survivors who were within 1–5 years of having a mastectomy from a nationwide cancer survivorship cohort. Physical and mental QOL, measured by SF-8, were also assessed. We translated, double-coded, and thematically analyzed transcripts according to QOL domains.

**Results:**

Over half of participants were diagnosed with stage III disease; all received chemotherapy and mastectomy. Key aspects of physical, financial, psychological, and social QOL that were influenced by treatment included the following: (1) pain, mobility, and fatigue; (2) reduced work capacity and financial stress; (3) depression, anxiety, and body image; and (4) mixed social support due to stigma. We also identified structural factors that limited access to symptom management and support services, which affected multiple aspects of QOL.

**Conclusions:**

Long-term pain, mobility restrictions, and fatigue affected physical functioning, specifically regarding employment, which had an overall negative impact on household financial status. Though some survivors received informal support through social networks, access to symptom management, support services, and public assistance was limited.

**Implications for Cancer Survivors:**

This study highlights multi-level challenges that affect QOL after mastectomy in Botswana. Interventions enhancing physical symptom management and improving psychosocial support services may improve QOL of survivors across sub-Saharan Africa.

## Introduction

Breast cancer is the most commonly diagnosed malignancy and leading cause of female cancer death worldwide [1]. The disease is increasingly affecting women in low-and-middle-income countries (LMICs) [2, 3], and mortality rates are particularly high in many sub-Saharan African (SSA) countries [4, 5]. In SSA, over three-quarters of women are diagnosed with advanced disease (i.e., stage III/IV), with geographic and socioeconomic disparities [6, 7]. Limited access to screening, diagnostic, and treatment services [8] contributes to low survival, with some estimating 5-year survival in SSA is approximately 40% [9, 10]. For those who receive treatment, mastectomy is often combined with chemo-, radio-, and hormonal therapies, which have profound impacts on survivors’ quality of life (QOL).

QOL is multidimensional, and breast cancer treatment can have adverse effects on physical, psychological, and social well-being [11]. For example, physical functioning post-surgery is worse for women undergoing modified radical mastectomy compared to breast-conserving surgery [12]. Women also experience poor body image, depression, and anxiety post-mastectomy [13–15], though these may vary over time and by demographic factors (e.g., age and marital status) [16]. Cultural stigmatization of breast cancer in SSA is a well-documented barrier to disclosing diagnosis with peers and engaging with cancer care [17–19]. With increasing breast cancer incidence in SSA, understanding survivors’ needs remains important for improving management and overall QOL, yet interventions for survivors and caregivers remain scant in SSA [20].

In Botswana, a middle-income country in SSA, early-stage breast cancer diagnosis is relatively rare due to limited access to early detection services. Over 65% of breast cancer patients are diagnosed with stage III disease [21, 22]. The majority (85%) of breast cancer patients in Botswana receive modified radical mastectomy over breast-conserving lumpectomy [23]. Though a national cancer control program including survivorship care is being developed [24], QOL following mastectomy remains understudied. Therefore, we explored breast cancer survivors’ experiences after treatment, especially related to mastectomy, to identify factors influencing QOL and opportunities to improve breast cancer survivorship in Botswana.

## Methods

### Study setting

In Botswana, some opportunistic screening has been offered by non-government organizations [25]; there is no formal, organized breast screening program. Patients presenting with symptoms at local clinics are referred for diagnostic workup at tertiary hospitals in the larger cities. Cancer diagnostics and treatment services are provided at no cost for citizens at public facilities. Although private hospitals offer services through a combination of out-of-pocket and medical insurance payments [26–28], the majority (83%) of the population relies on public coverage. In fact, over 90% of cancer patients rely on public facilities [29]. Patients may be treated with any combination of surgery, radio-, chemo-, or endocrine therapies, which are all publicly funded. At the time of study, public hospitals did not have the capacity to perform sentinel lymph node biopsies, so modified radical mastectomies were performed with axillary lymph node dissection. During the study period, radiotherapy was provided by a single private hospital and was subsidized by the government for all patients. Limited human resources, medication stockouts and equipment failures, and centralized oncology care are documented barriers to guideline-concordant breast cancer care [30, 31].

### Sampling and recruitment

Participants were identified and recruited from a prospective survivorship cohort—the Thabatse Cancer Cohort (TCC) [32–34]. Since 2010, the cohort has consented patients entering oncology care in Botswana to collect diagnosis, treatment, and outcomes data. During initial oncology visits, patients complete intake interviews, and their medical records are reviewed. Patients in the TCC are followed quarterly for 5 years.

For this qualitative study, potential participants were eligible if they were diagnosed with non-metastatic breast cancer, aged > 18 years, and underwent mastectomy within 1–5 years. Patients with metastatic disease were excluded in order to avoid heterogeneity in QOL implications due to treatment differences, poorer survival, and greater loss to follow-up [35]. Potential participants were contacted by phone to schedule an interview at their convenience. We purposefully recruited participants with a range of ages at diagnosis, urban and rural residences, and time since mastectomy.

### Data collection

Survivor demographics, HIV status, breast cancer characteristics, and patient-reported QOL measures were extracted from the TCC. Participants completed the Short Form-8 (SF-8) [36–39] approximately every 3 months as part of TCC. The questionnaire covers eight QOL domains and was previously validated in a Setswana-speaking population [40]. General health, physical functioning, bodily pain, and role physical items are included in the physical composite score (PCS), whereas vitality, social functioning, mental health, and role emotional make up mental composite scores (MCS). Scores range from 0 to 100, with higher scores indicating better QOL.

Based on a review of the literature and expert consensus, the semi-structured interview guide collected additional demographic information and focused on women’s QOL experiences post-mastectomy. Questions covered various QOL domains: physical functioning and symptoms, employment and financial status, emotional well-being and spirituality, and relationships. Some examples of questions included, “What symptoms are you experiencing? In what ways has the mastectomy affected your ability to get around? How did the treatment affect your ability to perform job? How has your breast cancer diagnosis impacted your personal and family finances? How have your relationships with family and friends changed since having the mastectomy?” We also specifically probed on additional services: “Have you ever used a breast prosthesis or something similar? What was your experience?” We conducted two pilot interviews before finalizing the guide.

A female native Setswana speaker (BM) conducted interviews with an English-speaking male (JRW) after being trained in qualitative methods. The interviewers had not previously interacted with participants through TCC, nor were they involved in patient care. All interviews were conducted in Setswana and took place in a quiet, private space—either in the participant’s residence or workplace (*n* = 16, 70%) or in a health facility office (*n* = 7, 30%) per participant preference. Informed consent was obtained before interviews began. Recordings were professionally transcribed directly into English and deidentified. Transcripts were not reviewed by participants. Interviews lasted 45 min on average.

We conducted interviews iteratively from February to April 2022. Data collection ended upon reaching data saturation, when no new themes were identified and QOL experiences became redundant [41]. This protocol was reviewed and approved by the Harvard T.H. Chan School of Public Health Institutional Review Board (Pro#18653) and the Health Research Development Committee of the Botswana Ministry of Health.

### Data analysis

Key themes from transcripts were labeled using a codebook informed by interview guide topics and SF-8 items. Codes were iteratively revised throughout the coding process and organized by QOL domains: (1) physical symptoms (e.g., pain and discomfort), (2) functioning (e.g., mobility and energy/fatigue), (3) financial well-being (e.g., work capacity and material resources), (4) psychological health (e.g., self-esteem, body image, and distress), and (5) social relationships (e.g., partners, family members, and social support). We also coded for social and structural context factors related to breast cancer care in Botswana that affected QOL and daily living (e.g., reliance on subsistence farming, accessibility of health services and supplies, and public assistance programs).

Study team members met routinely to discuss and determine code application in *Atlas.ti 23*. Transcript coders (JRW, BM, and RBW) met routinely to reconcile discrepancies from independent coding and reach consensus on definitions and concepts. Sixty-eight percent of transcripts were double-coded. Study team members (JRW, RBW, and REK) reviewed, analyzed, and summarized qualitative themes following an immersion-crystallization approach [42]. Throughout data collection and analysis, we employed multiple practices to evaluate potential interviewer, interview setting, and analyst influences and biases [43]. For example, interview summaries and coding memos included interviewer/reader/writer appraisals, and group discussions of these documents reflected on personal experiences, interpersonal issues, and contextual factors influencing our interpretations. The broader study team assisted with interpreting, corroborating, and synthesizing key findings. We report results according to COREQ guidelines [44].

We extracted SF-8 responses closest to the date of mastectomy and qualitative interview to calculate change for each sub-scale, PCS, and MCS. Mean time from surgery to SF-8 response was 120 days vs. mean of 29 days from qualitative interview. Descriptive statistics were used to summarize demographic and clinical characteristics and SF-8 scores.

Although this was a qualitative-driven analysis, we used a convergent mixed methods approach to integrate and merge data from both sources [45]. Quantitative variables (SF-8 domains, items) informed the initial coding framework, and quantitative responses were compared with qualitative narratives [46]. Data were integrated to examine patterns about how patient characteristics and themes affected changes in QOL. We jointly displayed themes-by-statistics to gain additional insights.

## Results

### Sample characteristics

Women (*n* = 23) were 55 years old on average (Table 1). Most survivors were either married/partnered (56%) and 30% had only completed primary school. Over three-quarters (78%) lived in a rural area (i.e., villages). Over one-third (39%) were living with HIV. Over half (57%) were diagnosed with stage III breast cancer and 74% had hormone receptor-positive disease. All participants received some oncology care at public facilities. No participants reported reconstructive surgery.

### Key thematic findings

Below we describe key themes of QOL domains (physical, financial, psychological, and social) that were affected by treatment: (1) pain, mobility, and fatigue; (2) capacity to work and financial stress; (3) depression, anxiety, and body image; and (4) stigma affecting social relationships. We identified additional structural and contextual factors that had broad, underlying influences on QOL, including health system factors (e.g., access to physiotherapy services and prosthetic supplies) and policy factors (e.g., formal employment and public assistance programs). Despite many reports of diminished QOL, support from family, neighbors, or friends helped many adjust and maintain QOL after treatment.

### Mixed methods data integration

Initially, using the SF-8 score closest to surgery, mean PCS was 51.5 and mean MCS was 47.0. For the post measures closest to the interview, PCS was 53.1 and mean MCS was 52.0. The lowest scoring sub-scale at both times was role emotional. Although mean PCS and MCS scores increased across the sample, we observed subgroup patterns of QOL implications (Table 2). Although there was some divergence (i.e., quantitative QOL changes did not always align with qualitative experiences), we identified patterns by QOL change (i.e., better or worse) across themes. Joint displays with illustrative quotes of QOL impacts due to treatment are presented by subtheme for physical QOL changes (Table 3) and psychological and social QOL (Table 4). Themes related to financial well-being influenced both physical and psychological QOL and are therefore included in both tables.

### Physical health

#### Pain, numbness, and swelling

A few survivors reported complications from surgery and extended recovery periods. For example, one participant described contralateral breast cancer and undergoing two consecutive mastectomies which caused her “excruciating pain.” Other participants’ surgical wounds took many months to heal, as one explained, because “they had to cut the rotten skin, it was infected twice.” These same participants recalled symptoms being downplayed by providers when they presented with long-term pain and swelling.

In fact, all but two participants experienced short- or long-term pain or swelling post-mastectomy. In the weeks following surgery, a few women described swelling and even more had “pain on the arm but no swelling” that subsided as they recovered. Fourteen women experienced persistent pain, numbness, and swelling in their arms and shoulders that “doesn’t go away…it’s always there,” lasting years for some. Consequently, participants took either over-the-counter or prescription (e.g., morphine) “pain killers” and muscle relaxers for long-term pain when it was “not subsiding.”

A few participants recalled being counseled about how swelling could occur after surgery “where they remove the glands” or that “water was not draining well” from surgical sites. However, many participants did not understand why they experienced particular symptoms and did not remember receiving such counseling. A few were prescribed compression sleeves to reduce lymphedema; however, they were unable to access them as these items were often out-of-stock at public facilities and pharmacies and had to be purchased privately. Others received instructions and recommendations for stretches and movement; two were prescribed physiotherapy sessions. However, physiotherapy appointments were not attended consistently.

#### Limited arm and shoulder mobility

Limited arm and shoulder mobility reduced survivors’ ability to perform activities of daily living (e.g., laundry, cooking, and farming) as they “used to do like a normal person” before mastectomy. Many survivors echoed “I can’t reach certain heights like I used to before” or “lift heavy objects” without feeling pain, so they instead “don’t touch anything that is stored very high.” Others reported switching to their non-dominant arms due to her operation. Many reiterated “when I need to cook and have to stir a pot, he [my partner] does all those” tasks.

#### Fatigue

Survivors recounted similar narratives about never fully recovering their energy and strength: “my life is difficult because I used to be able to plow at my field but now, I am no longer able to remove weeds.” As one described, “if you do hard labor, you get pain that requires you to just sleep.” Even less strenuous teaching activities were a struggle.

As a result, participants restricted daily activities to “make sure I take the right amount of work based on my body’s capability” to avoid exhaustion. This was discussed most in the context of daily chores, so some would “work smart, limiting what I can manage” and “take a break and continue again tomorrow.” In contrast, four women described how they “do the daily activities as usual,” such as cooking and laundry, and did not notice a decrease in their energy levels since treatment.

### Financial well-being

#### Reduced work capacity

Roughly half of the sample was employed at the time of interview. However, nearly two-thirds of women, especially those with young families, were unable to provide for their households after surgery, half of which was due to job loss. Some shared that employers offered financial support or sick leave for surgery, but the time away for treatment and recovery led to unemployment for others: “When I got sick, I lost my job.” A couple of women switched to lighter workloads at their workplace, but several reported changing jobs to find new, less labor-intensive jobs after recovering. Only a few participants considered their financial status largely unchanged post-surgery. Most of these women held positions with less physical demands (e.g., tailor), were retired, or received medical aid.

#### Financial stress despite support

Financial stress due to unemployment or not earning their pre-surgery income was common as women noted, “I have no money and the little I have is not enough.” Some older participants received financial support from their adult children. A few participants were briefly enrolled in a short-term unemployment relief program, *Ipelegeng*, that required beneficiaries to work on community projects for a small wage with monthly re-enrollment. Others received public welfare assistance or medical aid. However, program eligibility restricted simultaneous support from programs, and the assigned work was very “labor-intensive—they dig trenches, they cut trees,” which prohibited survivors’ participation. Despite receiving medical aid, participants remained worried it would end “at any time and tell me they will no longer be helping me. And I wonder what I will do then as I can’t do anything now?”

### Psychological health

#### Depression and anxiety eased over time

Several participants reported feeling particularly low, “really down,” or anxious immediately after surgery as they grieved the loss of their breast. However, grief and depressive symptoms usually improved as they physically recovered, processed their loss, and adjusted to their new appearance. The lingering anxiety and worries participants had were about fear of recurrence, cancer risk for their children, uncertainty about the future, and stress from severe pain. For example, distress stemmed from intrusive thoughts about their situation and the future as well as being hyper-sensitive about symptoms which provoked recurrence concerns. Some worried about familial risk for their daughters and relatives, who had also become anxious about developing breast cancer.

Others seemed to have adjusted more easily. For example, a few participants were “not completely happy” since their mastectomies but considered themselves “just fine.” In fact, ten reported little to no psychological impact by the time of the qualitative interview explaining they “hardly feel stressed.” For some, relying on their faith and spiritual relationships helped them cope with distress. Religious beliefs, “read[ing] the Bible,” and praying helped ease their fears “so that I don’t get any anxiety.”

#### Loss of confidence in body image and femininity

Participants expressed conflicting feelings about being insecure about their bodies, accepting themselves, worrying about how their physical appearance is perceived, and choosing not to lament over the opinions of others. Due to negative body image, some explained how they dressed differently than prior to surgery, wearing loose clothing that covers more, using safety pins to conceal their missing breast so others would not notice. Multiple women talked about feeling “abnormal […] it will never be the same as before” and “not the same as other people” so “you lose some confidence.” This left some still feeling “very sad” and “always on the lookout,” especially about bathing and dressing. Though others shared how “talk[ing] a lot about my health” led to them eventually feeling comfortable enough to undress in front of relatives again.

The majority of participants obtained either a knitted or silicone prosthetic bra insert. Most were offered a “woolen” insert from the public hospital or charity foundation. Roughly half received a free silicone insert from the hospital, though some made commercial purchases. However, others reported limited access due to prosthetics being out of stock in their size, high commercial costs, and transportation challenges associated with rurality. At the time of their interviews, four participants were still waiting for a prosthetic. These women reported using pantyhose and “anything accessible or available: a towel, a scarf,” while waiting.

Four women were dissatisfied with knitted prosthetics and another eight noted discomfort with silicone prosthetics. Silicone was described as “not the same size as the natural breast” and many reported both as “uncomfortable,” explaining how the prosthetics “shrink” in the wash, wear out quickly, feel “too heavy” and sometimes “dislodge.” Others described, “if it’s warm, it sweats and then becomes sticky and […] uncomfortable.”

### Social relationships

#### Mixed social support due to stigma

In a few cases, family members, friends, and partners had mixed reactions and abandoned survivors after mastectomy. For one participant, her friends “would be uncomfortable” around her at first, and then “became distant” because “they don’t want to be around a person who doesn’t have a breast.” Similarly, one participant’s partner “used to be there before” but had since left the relationship, and another “ended up moving out of the home” because “they didn’t treat me well.” However, one participant spoke of her children being supportive and protective as they too worried about stigma and encouraged her not to disclose her diagnosis.

Others did not feel stigmatized: “we’re still living the same as we used to.” Family members, friends, partners, and co-workers were described as being supportive, which improved survivors’ perspectives on their prognosis. For example, most participants reported that family members provided emotional support, were “by my side from day one until today,” or “with me all the way.” Many women credited their physical and mental health to the spiritual support they received from friends who prayed with them, offered encouragement, and would “check on me.”

## Discussion

This analysis identified physical, financial, psychological, and social QOL implications after breast cancer surgery among survivors in Botswana. These findings provide key insights to inform survivorship interventions focused on enhancing physical symptom management and improving psychosocial support services. Pain and mobility limitations affected activities of daily living, and few were able to return to work; thus, financial well-being diminished. Although families and social networks were mostly supportive, depression and body image concerns were closely tied to social stigma; a few survivors were abandoned due to mastectomy. Structural factors like unemployment program policies and limited access to medication and services affected multiple QOL domains, especially for rural residents.

Persistent symptoms including arm and shoulder pain, swelling, numbness, and fatigue were common in our sample, which may explain the minimal increase in PCS over time. Additionally, limited mobility decreased functional capacity for household chores such as obtaining water, cooking, and cleaning. In Botswana and other SSA countries with rural populations or reliance on subsistence farming [47, 48], diminished QOL due to these symptoms is particularly debilitating for women diagnosed with advanced disease or with lower socioeconomic status [49, 50]. The mixed PCS changes may be due to functional recovery immediately post-treatment, though long-term disability remains a challenge as demonstrated in a larger quantitative analysis of breast cancer survivors in the TCC [51]. Functional loss and fatigue were linked with nearly all participants’ individual and household financial well-being as many were no longer able to earn livable wages. This cycle of reduced physical function or fatigue interfering with employment and ultimately financial hardship is well-documented across SSA [19, 52–54], which may also increase risk of depression [55].

We also observed psychological and social changes affecting breast cancer survivors’ QOL, largely due to depression, anxiety, and stigma immediately after treatment. Like findings from Ghana and Ethiopia [14], some of our participants saw improvements in MCS over time as they coped with and accepted their new appearance and limitations. Negative body image and stigma were persistently problematic for other participants in our study, which may also explain the persistently low role emotional sub-scale. Others have similarly found that distress from bodily changes was linked to depression [56]. Like others, we also found that a supportive social network was key to coping, though dressing differently and packing bras also helped [57, 58]. Notably, post-mastectomy reconstructive and plastic surgery only became available in Botswana in 2022 [59]; thus, it was not an option for any study participants. Moving forward, research on access to, utilization of, and preferences for these services will be important.

Interpersonal relationships with children, siblings, friends, and partners were sometimes negatively affected due to the stigmatization of breast removal and cancer generally. Similar narratives of mastectomy and/or cancer stigma have been reported in Malawi [19]. Navigation programs, such as social workers being more involved with diagnostic counseling or peer-led groups, may help patients and their families better anticipate what to expect after surgery and treatment [20, 60–62]. Still, the majority of survivors in our study had supportive family members and friends that provided encouragement, assisted with household chores, and offered financial backing. Faith-based relationships were particularly helpful for maintaining or improving psychological and social QOL, which is well documented among cancer survivors across SSA [63–66]. Taken together, these sub-themes help explain the overall MCS gains we observed. Incorporating spiritual beliefs into education and navigation may help reinforce emotional and social QOL to improve survivor experiences [67].

We also observed structural and social determinants of health (i.e., access to care, economic stability, and built environment) that had cross-cutting implications on QOL [11]. Access to medication, approaches to prevent and mitigate lymphedema-related disability, and adequate breast prosthetics were suboptimal. Transportation challenges and rurality, which are well-documented across SSA [68, 69], exacerbated limited service availability, which may have driven the subgroup differences we observed. Additional research on post-axillary node dissection morbidity and lymphedema rates is needed as they have not yet been well researched in the region and may help identify those at risk of poor QOL. Still, these findings highlight the need for expanded access to post-surgery care, including physiotherapy services and compression garments for managing physical symptoms. And although some women received public assistance, eligibility restrictions and physical labor requirements were problematic. Indeed, few cancer control plans across SSA include strategies related to employment concerns [70]. Expanding coverage for public assistance programs and tailoring working conditions to survivors’ short- and long-term mobility limitations may help subgroups with poor physical QOL and also minimize downstream effects of job loss.

These findings must be interpreted in light of some limitations. Because the SF-8 data were extracted from a broad cohort database that followed multiple types of cancer patients, they may not capture breast cancer specific QOL implications, such as lymphedema. Additionally, the timing of QOL assessments may influence the findings, given the variation in time between SF-8 and surgery dates and time between surgery and interview dates. Although we focused on mastectomy-related influences on QOL, some symptoms, like fatigue, may result from a combination of surgery and other treatments (i.e., chemo-, radio-, and hormonal therapies). Despite these limitations, to our knowledge, this is the first qualitatively driven mixed methods exploration of QOL after breast cancer surgery and in Botswana.

## Conclusions

This analysis provides important insights about physical, financial, psychological, and social setbacks affecting breast cancer survivors’ QOL. These findings underscore the need for multi-level strategies to improve survivorship care in Botswana, including expanding access to symptom management, support services, and public assistance programs.

## Figures and Tables

**Table 1:** Sample characteristics of female breast cancer interview participants

	*N* (%) 23 (100)
**Mean age at diagnosis (SD)**	48.9 (10.1)
**Educational attainment**	
Primary	7 (30.4)
Junior secondary	8 (34.8)
Senior secondary	3 (13.0)
Tertiary	5 (21.7)
**Marital status**	
Married/partnered	13 (56.5)
Not married	8 (35.3)
Widowed	1 (4.3)
Missing	1 (4.3)
**Employment status**	
Full time	11 (47.8)
Not currently employed including retired	11 (47.8)
Missing	1 (4.3)
**Residence**	
Rural village	18 (78.3)
Urban town/city	5 (21.7)
**HIV status**	
HIV-positive	9 (39.1)
HIV-negative	14 (60.8)
**Breast cancer stage**	
I/II	10 (43.5)
III	13 (56.5)
**Hormone receptor status**	
HR-positive	17 (73.9)
HR-negative	6 (26.1)
**Treatment received**	
Chemotherapy	23 (100.0)
Radiation therapy	19 (82.6)
Endocrine therapy	17 (73.9)
**Mean years since surgery (SD)**	3.5 (0.65)

**Table 2:** Breast cancer survivor SF-8 QOL score changes from treatment to interview date*

QOL impact	# survivors	PCS change	MCS change
↑ PCS ↑ MCS	8	10.00	14.31
↓ PCS ↓ MCS	5	−7.66	−8.14
↑ PCS ↓ MCS	4	4.56	−4.77
↓ PCS ↑ MCS	6	−3.61	10.23
Overall	23	1.67	5.05

*PCS* physical composite score, *MCS* mental composite score; ↑, increase/better; ↓, decrease/worse

* Mean 3.5 years between interview and surgery

**Table 3:** Exemplary quotes of physical QOL changes by theme

SF-8 PCS change	Key theme
Pain numbness, and swelling	
Worse PCS	Even now [2 years later] the arm is still swollen. Pain is still there. It also feels tight. The shoulder works ok. But the arm as a whole doesn’t work […] It doesn’t do anything heavy, most of the time when I lift things—like fetching water from the outside tap—I use my left arm – Stage III, 56 years
Little/no change PCS	I was moving it up like this so that it won’t refuse to move. It has never gotten swollen. They [medical team] said I should use it [arm] and stretch it. Sometimes it can be stuck like that for good if not moved – Stage II, 40 years
Limited arm and shoulder mobility	
Worse PCS	I cannot wash or scrub the pots, my daughter does it for me, washing is done by my daughter or one of my friends helping my daughter – Stage II, 65 years
Little/no change PCS	There is pain, but it’s not all the time […] when I’m sitting like this, I can feel pain here. And the scar can get a bit itchy. [But] when I bathe, I can lift it well. It doesn’t restrict me to do anything. Only the arm swells. […] when I use it too much, it swells – Stage III, 55 years
Fatigue	
Worse PCS	It has affected me a lot when it comes to household activities. And even in school: we write on the board, we mark—I am unable to lift up my arm for a longer time. I feel tired – Stage II, 54 years
Little/no change PCS	I don’t work like I did before. Most of the time I limit the amount of work—I don’t’ want to overwork. I make sure I take the right amount of work based on my body’s capability – Stage III, 40 years
Reduced capacity to work	
Worse PCS	It was like our life was going down because I no longer had money […] As I no longer work, there is hunger. So, I should be assisted with the “basket,” and go to the social welfare people so that I can be assisted – Stage III, 64 years
Little/no change PCS	They [employer] work with me knowing that I can’t do some of the things I used to do before the operation, I have had no issues or conflicts with them – Stage I, 72 years

**Table 4:** Exemplary quotes of psychological and social QOL changes by theme

SF-8 MCS change	Key theme
Financial stress	
Worse MCS	My anxiety is because I wonder if I were to get a job, will I be able to do it? Will I be able to provide for my children? I don’t know what the future holds for me – Stage III, 50 years
Little/no change MCS	It didn’t affect the family or work as I continued to work. I was entitled, every worker is allowed to have sick leave if granted by the medical team, to stay at home and take care of themselves – Stage II, 45 years
Depression and anxiety	
Worse MCS	Feeling pity for myself, wondering if I am dying […] ‘why is it like I am going to suffer? Why can’t I just die once than suffer because of persistent pains?’ – Stage III, 63 years
Little/no change MCS	Your feelings go down if you don’t have people who support you. If you have supportive people at home and externally, you relax because you can see that you’ve got a family – Stage III, 61 years
Body image concerns	
Worse MCS	But as a woman, even if other people cannot realize you don’t have a breast, you personally will always think that even when you are dressed, you are still abnormal. When you have removed the breast, it will never be the same as before […] You lose some confidence – Stage II, 52 years
Little/no change MCS	I thought he [partner] would run away when seeing me with one breast […] That was one thing I was very wary about and thought he may be forcing himself to stay with me, but he has proved that he is here for me – Stage II, 45 years
Mixed social support due to stigma	
Worse MCS	There was no one checking on me […] After I was discharged from the hospital, they [siblings] came to help me with my luggage from the hospital and went and dumped me where I was renting. And they went for good. I really struggled […] My son was scared of me. There was nothing I could do […] he went away. I was staying here on my own – Stage II, 39 years
Little/no change MCS	They helped me to overcome that thought and I am now free […] initially I had not accepted myself, I hid and did not disclose – Stage III, 67 years

## Data Availability

The datasets generated during and/or analyzed during the current study are available from the corresponding author on reasonable request. No datasets were generated or analysed during the current study.

## References

[1] Sung H, Global cancer statistics 2020: GLOBOCAN estimates of incidence and mortality worldwide for 36 cancers in 185 countries. CA Cancer J Clin. 2021;71(3):209–49. 10.3322/caac.21660 33538338

[2] Arnold M, Current and future burden of breast cancer: global statistics for 2020 and 2040. Breast. 2022;66:15–23. 10.1016/j.breast.2022.08.010 PMC9465273 36084384

[3] Francies FZ, Breast cancer in low-middle income countries: abnormality in splicing and lack of targeted treatment options. Am J Cancer Res. 2020;10(5):1568–91. PMC7269781 32509398

[4] Bray F, Parkin DM. Cancer in sub-Saharan Africa in 2020: a review of current estimates of the national burden, data gaps, and future needs. Lancet Oncol. 2022;23(6):719–28. 10.1016/S1470-2045(22)00270-4 35550275

[5] Ferlay JEM, Lam F, Laversanne M, Colombet M, Mery L, Piñeros M, Global cancer observatory: Cancer today Lyon. France: International Agency for Research on Cancer; 2024. Available from: https://gco.iarc.who.int/today.

[6] Jedy-Agba E, Stage at diagnosis of breast cancer in subSaharan Africa: a systematic review and meta-analysis. Lancet Glob Health. 2016;4(12):e923–35. 10.1016/S2214-109X(16)30259-5 PMC5708541 27855871

[7] Vanderpuye V, Assessment of breast cancer management in sub-Saharan Africa. JCO Glob Oncol. 2021;7:1593–601. 10.1200/GO.21.00282 PMC8624034 34843373

[8] Twahir M, Real-world challenges for patients with breast cancer in sub-Saharan Africa: a retrospective observational study of access to care in Ghana, Kenya and Nigeria. BMJ Open. 2021;11(3):e041900. 10.1136/bmjopen-2020-041900 PMC7929861 33653746

[9] Limenih MA, Survival patterns among patients with breast cancer in sub-Saharan Africa: a systematic review and meta-analysis. JAMA Netw Open. 2024;7(5):e2410260. 10.1001/jamanetworkopen.2024.10260 PMC11094564 38743426

[10] World Health Organization. Addressing inequities in breast cancer treatment in sub-Saharan Africa: insights from a breast cancer surgeon in Nairobi. 2022. Available from: https://www.who.int/news-room/feature-stories/detail/addressing-inequities-in-breast-cancertreatment-in-sub-saharan-africa--insights-from-a-breast-cancer-surgeon-in-nairobi.

[11] Qan’ir Y, Quality of life among patients with cancer and their family caregivers in the sub-Saharan region: a systematic review of quantitative studies. PLoS Glob Public Health. 2022;2(3):e0000098. 10.1371/journal.pgph.0000098 PMC10021310 36962119

[12] Senoga A, Quality of life of patients one year after breast-conserving surgery versus modified radical mastectomy for early breast cancer: a Kenyan tertiary hospital five-year review. Pan Afr Med J. 2023;46:69. 10.11604/pamj.2023.46.69.39151 PMC10822102 38282779

[13] Javan Biparva A, Global depression in breast cancer patients: systematic review and meta-analysis. PLoS ONE. 2023;18(7):e0287372. 10.1371/journal.pone.0287372 PMC10370744 37494393

[14] Kennedy SH, A prospective evaluation of the quality of life and mental health implications of mastectomy alone on women in sub-Saharan Africa. Ann Surg. 2023;278(5):e1080–6. 10.1097/SLA.0000000000005891 37144388

[15] Wondimagegnehu A, Depression and social support among breast cancer patients in Addis Ababa, Ethiopia. BMC Cancer. 2019;19(1):836. 10.1186/s12885-019-6007-4 PMC6712811 31455282

[16] Abebe E, Female breast cancer patients, mastectomy-related quality of life: experience from Ethiopia. Int J Breast Cancer. 2020;2020:8460374. 10.1155/2020/8460374 PMC7171616 32328310

[17] Martei YM, Stigma and social determinants of health associated with fidelity to guideline-concordant therapy in patients with breast cancer living with and without HIV in Botswana. Oncologist. 2023. 10.1093/oncolo/oyad183. PMC10712728 37405697

[18] Sakafu LL, Delayed diagnostic evaluation of symptomatic breast cancer in sub-Saharan Africa: a qualitative study of Tanzanian women. PLoS ONE. 2022;17(10):e0275639. 10.1371/journal.pone.0275639 PMC9536581 36201503

[19] Watt MH, Cancer-related stigma in Malawi: narratives of cancer survivors. JCO Glob Oncol. 2023;9: e2200307. 10.1200/GO.22.00307 PMC10166375 36795989

[20] Guan T, Systematic review of psychosocial interventions for adult cancer patients and their family caregivers in sub-Saharan Africa. Glob Public Health. 2023;18(1):2199062. 10.1080/17441692.2023.2199062 PMC10623887 37054448

[21] Sinha S, Epidemiology of breast cancer presentation in Botswana, South Africa, and the United States. J Surg Res. 2022;279:533–9. 10.1016/j.jss.2022.04.071 PMC10033457 35868037

[22] Bhatia RK, Report of clinico-pathological features of breast cancer in HIV-infected and uninfected women in Botswana. Infect Agents Cancer. 2019;14(1):28. 10.1186/s13027-019-0245-6 PMC6805363 31649747

[23] Wester J, Clinical characteristics and outcomes of breast cancer in Botswana. JCO Glob Oncol. 2022;8(Supplement_1):35–35.

[24] Makoni M. Botswana plans first-ever national cancer control programme. Lancet Oncol. 2023;24(2):127. 10.1016/S1470-2045(22)00784-7 36566759

[25] Dykstra M, Impact of community-based clinical breast examinations in Botswana. JCO Glob Oncol. 2021;7:17–26. 10.1200/GO.20.00231 PMC8081526 33405960

[26] Ministry of Health. Towards a Healthier Botswana. Gaborone, Botswana: Ministry of Health; 2012.

[27] Ministry of Health and Wellness. Our health care system. [cited 2025; Available from: https://www.moh.gov.bw/health_care.html#. Accessed 5/5/2025.

[28] Tapera R, Moseki S, January J. The status of health promotion in Botswana. J Public Health Afr. 2018;9(1):699. 10.4081/jphia.2018.699 PMC6057722 30079160

[29] Suneja G, Cancer in Botswana: resources and opportunities. Lancet Oncol. 2013;14(8):e290–1. 10.1016/S1470-2045(13)70283-3 23816293

[30] Ralefala T, Provider barriers and facilitators of breast cancer guideline-concordant therapy delivery in Botswana: a consolidated framework for implementation research analysis. Oncologist. 2021;26(12):e2200–8. 10.1002/onco.13935 PMC8649035 34390287

[31] Martei YM, Availability of WHO essential medicines for cancer treatment in Botswana. J Glob Oncol. 2018;4:1–8. 10.1200/JGO.17.00063 PMC6223417 30241225

[32] Dryden-Peterson S, Hiv infection and survival among women with cervical cancer. J Clin Oncol. 2016;34(31):3749–57. 10.1200/JCO.2016.67.9613 PMC5477924 27573661

[33] Iyer HS, Explaining disparities in oncology health systems delays and stage at diagnosis between men and women in Botswana: a cohort study. PLoS ONE. 2019;14(6):e0218094. 10.1371/journal.pone.0218094 PMC6553768 31170274

[34] Moahi K, HIV and Hodgkin lymphoma survival: a prospective study in Botswana. JCO Glob Oncol. 2022;8: e2100163. 10.1200/GO.21.00163 PMC8769145 35025689

[35] Joko-Fru WY, Breast cancer survival in sub-Saharan Africa by age, stage at diagnosis and human development index: a population-based registry study. Int J Cancer. 2020;146(5):1208–18. 10.1002/ijc.32406 PMC7079125 31087650

[36] Aoki M, Autonomic function measurements for evaluating fatigue and quality of life in patients with breast cancer undergoing radiation therapy: a prospective longitudinal study. Radiat Oncol. 2023;18(1):171. 10.1186/s13014-023-02362-w PMC10585884 37858146

[37] Arinaga Y, The 10-min holistic self-care for patients with breast cancer-related lymphedema: pilot randomized controlled study. Tohoku J Exp Med. 2019;247(2):139–47. 10.1620/tjem.247.139 30799328

[38] Mehnert A, Fear of cancer progression and cancer-related intrusive cognitions in breast cancer survivors. Psychooncology. 2009;18(12):1273–80. 10.1002/pon.1481 19267364

[39] Treanor C, Donnelly M. A methodological review of the short form health survey 36 (SF-36) and its derivatives among breast cancer survivors. Qual Life Res. 2015;24(2):339–62. 10.1007/s11136-014-0785-6 25139502

[40] Onagbiye SO, Moss SJ, Cameron M. Validity and reliability of the Setswana translation of the Short Form-8 health-related quality of life health survey in adults. Health SA. 2018;23:1092. 10.4102/hsag.v23i0.1092 PMC6917454 31934383

[41] Saunders B, Saturation in qualitative research: exploring its conceptualization and operationalization. Qual Quant. 2018;52(4):1893–907. 10.1007/s11135-017-0574-8 PMC5993836 29937585

[42] Crabtree BF, Miller WL. Doing qualitative research. Third ed. Thousand Oaks, CA, USA: SAGE Publications, Inc; 2022.

[43] Olmos-Vega FM, A practical guide to reflexivity in qualitative research: AMEE guide no. 149. Med Teach. 2023;45(3):241–51. 10.1080/0142159X.2022.2057287 35389310

[44] Tong A, Sainsbury P, Craig J. Consolidated criteria for reporting qualitative research (COREQ): a 32-item checklist for interviews and focus groups. Int J Qual Health Care. 2007;19(6):349–57. 10.1093/intqhc/mzm042 17872937

[45] Guetterman TC, Fetters MD, Creswell JW. Integrating quantitative and qualitative results in health science mixed methods research through joint displays. Ann Fam Med. 2015;13(6):554–61. 10.1370/afm.1865 PMC4639381 26553895

[46] Love HR, Corr C. Integrating without quantitizing: two examples of deductive analysis strategies within qualitatively driven mixed methods research. J Mixed Methods Res. 2022;16(1):64–87.

[47] Kramer N, Ramjith J, Shamley D. Prevalence of shoulder morbidity after treatment for breast cancer in South Africa. Support Care Cancer. 2019;27(7):2591–8. 10.1007/s00520-018-4540-3 30456720

[48] Wuraola F, Prevalence and determinants of lymphedema in newly diagnosed Nigerian breast cancer patients using bioimpedance estimations. Ecancermedicalscience. 2023;17:1506. 10.3332/ecancer.2023.1506 PMC10129379 37113722

[49] Swart NC, Relationship between quality of life and symptom burden in patients with cancer with and without HIV in Botswana. Clin J Oncol Nurs. 2023;27(1):98–103. 10.1188/23.CJON.98-103 37677826

[50] Boucheron P, Self-reported arm and shoulder problems in breast cancer survivors in Sub-Saharan Africa: the African breast cancer-disparities in outcomes cohort study. Breast Cancer Res. 2021;23(1):109. 10.1186/s13058-021-01486-9 PMC8611842 34819118

[51] Dykstra MP, Quality of life gain following treatment among breast cancer survivors with and without HIV. JCO Glob Oncol. 2024;10: e2400110. 10.1200/GO.24.00110 PMC11315356 39116360

[52] Schantz C, Access to oncology care in Mali: a qualitative study on breast cancer. BMC Cancer. 2024;24(1):81. 10.1186/s12885-024-11825-6 PMC10788985 38225594

[53] Folorunso SA, Sociodemographic and treatment-related correlates of fatigue in breast cancer survivors at an oncology clinic in Nigeria. Ecancermedicalscience. 2024;18: 1659. 10.3332/ecancer.2024.1659 PMC10901630 38425762

[54] Khajoei R, Breast cancer survivorship needs: a qualitative study. BMC Cancer. 2024;24(1):96. 10.1186/s12885-024-11834-5 PMC10795302 38233789

[55] Mohammed A, Prevalence and associated factors of depression among breast cancer patients in sub-Saharan Africa: a systematic review and meta-analysis. SAGE Open Med. 2024;12:20503121241226897. 10.1177/20503121241226897 PMC10826378 38292418

[56] Kagee A, Roomaney R, Knoll N. Psychosocial predictors of distress and depression among South African breast cancer patients. Psychooncology. 2018;27(3):908–14. 10.1002/pon.4589 29178147

[57] Iddrisu M, Aziato L, Ohene LA. Socioeconomic impact of breast cancer on young women in Ghana: a qualitative study. Nurs Open. 2021;8(1):29–38. 10.1002/nop2.590 PMC7729654 33318809

[58] Olasehinde O, Life without a breast: exploring the experiences of young Nigerian women after mastectomy for breast cancer. J Glob Oncol. 2019;5:1–6. 10.1200/JGO.18.00248 PMC6550027 31095453

[59] Ramasu Phemelo. Dr. Tjinjeka - One of Two Local Plastic and Reconstructive Surgeons. Botswana Guardian [Internet]. 2022. Available from: https://www.pressreader.com/botswana/botswana-guardian/20220211/281642488587615.

[60] Pierz AJ, A scoping review: facilitators and barriers of cervical cancer screening and early diagnosis of breast cancer in Sub-Saharan African health settings. Gynecol Oncol Rep. 2020;33:100605. 10.1016/j.gore.2020.100605 PMC7327246 32637528

[61] Chidebe RCW, “Not even my husband knows that I have this [breast cancer]”: survivors’ experiences in accessing, navigating and coping with treatment. Support Care Cancer. 2024;32(2):112. 10.1007/s00520-024-08316-6 PMC10796523 38236480

[62] Adam A, Koranteng F. Availability, accessibility, and impact of social support on breast cancer treatment among breast cancer patients in Kumasi, Ghana: a qualitative study. PLoS ONE. 2020;15(4):e0231691. 10.1371/journal.pone.0231691 PMC7162460 32298340

[63] Nkoana S, Cancer survivorship: religion in meaning making and coping among a group of Black prostate cancer patients in South Africa. J Relig Health. 2022;61(2):1390–400. 10.1007/s10943-021-01406-3 PMC8967772 34468928

[64] Teshome MH, The lived experience of Ethiopian women after mastectomy due to breast cancer: a qualitative study. Asian Pac J Cancer Prev. 2024;25(1):103–8. 10.31557/APJCP.2024.25.1.103 PMC10911715 38285773

[65] Teye-Kwadjo E, Goka A-S, Ussher YAA. Unpacking the psychological and physical well-being of Ghanaian patients with breast cancer. Dialogues Health. 2022;1: 100060. 10.1016/j.dialog.2022.100060 PMC10953980 38515885

[66] Weru JK, Nafula EW, Silbermann M, Berger A. Psychosocial-Spiritual Healing: An Impression of the Impact of Culture and Faith in Cancer Care in Africa, Kenya, Sub-saharan Africa, Culture, Beliefs, Traditional Healers, Herbal Treatment, Religion, Spirituality, Ethnic Groups, Ancestors. Global Perspectives in Cancer Care: Religion, Spirituality, and Cultural Diversity in Health and Healing: Oxford University Press; 2022. p. 296–9.

[67] Martei YM, Development, acceptability and usability of culturally appropriate survivor narrative videos for breast cancer treatment in Botswana: a pilot study. BMJ Open. 2024;14(1):e073867. 10.1136/bmjopen-2023-073867 PMC10828869 38296302

[68] Kim J, Geospatial disparities in survival of patients with breast cancer in sub-Saharan Africa from the African breast cancer-disparities in outcomes cohort (ABC-DO): a prospective cohort study. Lancet Glob Health. 2024. 10.1016/S2214-109X(24)00138-4. PMC11168938 38788756

[69] Patel S, Expanding radiotherapy access in sub-Saharan Africa: an analysis of travel burdens and patient-related benefits of hypofractionation. Front Oncol. 2023;13:1136357. 10.3389/fonc.2023.1136357 PMC10151787 37143940

[70] Garton EM, An analysis of survivorship care strategies in national cancer control plans in Africa. J Cancer Surviv. 2023;17(3):634–45. 10.1007/s11764-022-01320-x 36656300

